# Characterization and co-expression analysis of WRKY orthologs involved in responses to multiple abiotic stresses in Pak-choi (Brassica campestris ssp. chinensis)

**DOI:** 10.1186/1471-2229-13-188

**Published:** 2013-11-25

**Authors:** Jun Tang, Feng Wang, Zhen Wang, Zhinan Huang, Aisheng Xiong, Xilin Hou

**Affiliations:** 1 State Key Laboratory of Crop Genetics and Germplasm Enhancement, College of Horticulture, Nanjing Agricultural University, Nanjing 210095, China

**Keywords:** WRKY transcription factor, Abiotic stress, Co-expression analysis, Subcellular localization, Pak-choi

## Abstract

**Background:**

The WRKY transcription factor is an important member of the stress-related transcription factors, which mediate diverse abiotic stresses in many plants. However, up until now, the number of WRKY members, and the regulatory mechanisms involved in abiotic stress responses in Pak-choi (*Brassica campestris* ssp. *chinensis*), remained unknown.

**Results:**

We isolated and identified 56 full-length WRKY cDNAs from a Pak-choi stress-induced cDNA library. The 56 putative BcWRKY proteins were divided into three groups based on structural and phylogenetic analyses. A subcellular localization prediction indicated that the putative BcWRKY proteins were enriched in the nuclear region. Experiments involving *BcWRKY25* and *BcWRKY40* confirmed the prediction. A total of 22 *BcWRKY*s were differentially expressed in response to at least one stress condition (abscisic acid, cold, salinity, heat, or osmosis) tested on Pak-choi leaves, and a co-expression analysis indicated stress-inducible *BcWRKY*s co-regulated multiple abiotic stresses. *BcWRKY33*, *BcWRKY40*, *BcWRKY53*, and *BcWRKY70* acted as key regulators and played dominant roles within co-regulatory networks of stress-inducible *BcWRKY*s.

**Conclusions:**

We first isolated and characterized the 56 stress-inducible WRKY transcription factor family members. A total of 22 stress-inducible *BcWRKY*s found in leaves can co-regulate multiple environmental stresses by integrating the potential mutual interactions of WRKYs in Pak-choi. This information will be valuable when exploring the molecular mechanisms of WRKYs in response to abiotic stresses in plants.

## Background

To overcome environmental stresses, plants have developed the ability to perceive and respond to these diverse external signals using specialized physiological and biochemical strategies [1,2]. Plant stress responses are generally controlled by a network of specialized genes that are intricately regulated by specific transcription factors (TFs) [3]. The WRKY TFs are important members of the stress-related TFs involved in regulating the plant’s environmental stress responses [4-6]. The WRKY TF family was named based on the presence of a 60 amino acid (aa) WRKY domain that is defined by a highly conserved WRKYGQK heptapeptide at the N-terminus and a zinc-finger-like motif at the C-terminus. WRKY proteins can be classified into three groups (I, II, and III) based on the number of WRKY domains and the pattern of the zinc-finger motif [5,7].

The WRKY TF family is a large conserved family of TFs that has been reported in many plants [8-15]. WRKY TFs have been found to be responsive to various abiotic stresses, including salinity, drought, cold, heat, and abscisic acid (ABA) signaling [16-20]. For instance, the NaCl-inducible *AtWRKY25* and *AtWRKY33* mediate abiotic stresses [21]. *AtWRKY63* is involved in plant responses to ABA and drought tolerance [22], and *AtWRKY34* mediates the cold sensitivity of mature pollen in *Arabidopsis*[23]. WRKY40, WRKY18, and WRKY60 interact with ABAR and negatively regulate ABA signaling [17]. Alleles of *OsWRKY45-1* and *OsWRKY45-2* play different roles in ABA signaling and salt stress tolerance in rice [24]. WRKY8 antagonistically interacts with VQ9 to modulate salinity stress tolerance [25]. Additionally, *BcWRKY46*, a novel cold-inducible gene from Pak-choi (*B. campestris* ssp. *chinensis*, synonym of *B. rapa* ssp. *chinensis*) enhances the cold, salt and dehydration stress tolerance in transgenic tobacco [26].

Because of the functional complexity of the WRKY genes involved in environmental stresses, many approaches have been used to explore the unknown mechanisms of the stress response processes. Previous studies have demonstrated the power of co-expression analysis as a candidate discovery tool [27-29], which encouraged us to explore this approach for the identification of genes putatively involved in these interesting biological processes. Additionally, a co-expression analysis of *OsWRKY*s under biotic and abiotic stress conditions has been reported [30].

Pak-choi is an important *Brassica* crop with exceptional cold resistance [31], and its nearest genetic relative that has been sequenced is Chinese cabbage (*B. rapa* ssp. *pekinensis*) [32], which provides an effective model for Pak-choi research. Although WRKYs have been reported to mediate various stresses, the number of WRKY members in Pak-choi and their roles in response to abiotic stress tolerance were still unknown. Here, we cloned and identified 56 stress-inducible WRKY orthologs from Pak-choi, and we systematically investigated their organization, subcellular localization, and expression patterns under multiple abiotic stresses. In addition, we simultaneously measured co-expression of the stress-inducible WRKY orthologs in Pak-choi and *Arabidopsis thaliana*. We subsequently established a co-regulatory network of stress-inducible *BcWRKY*s to multiple abiotic stresses, and indicated the possible interactions of stress-inducible *BcWRKY* gene pairs.

## Results

### Cloning stress-inducible BcWRKY genes from Pak-choi

We isolated 56 *BcWRKY* genes from a multiple abiotic stress-treated Pak-choi cDNA library using a homology cloning method, which was based on sequence information from the *A. thaliana* WRKY gene family and the Chinese cabbage *chiifu* genome. We first designed degenerate and oligo (dT) primers to amplify the conserved regions of WRKY orthologs. Based on the PCR products’ sequencing results, we designed gene-specific primers (Additional file 1: Table S1) and performed 5′-and 3′-RACE to amplify the full-length cDNA sequences of *BcWRKY*s from the stress-induced Pak-choi cDNA library. The stress-inducible Pak-choi WRKY genes ranged from 531 to 3,195 base pairs (bp) and included 56 ORFs. These ORFs were confirmed by sequencing, and the sequences were submitted to GenBank (Table 1). The *BcWRKY* genes were named based on their similarity to the *AtWRKY* orthologs, and the molecular properties and sequence characteristics of the putative BcWRKY proteins were also analyzed. Among the 56 BcWRKY proteins, the isoelectric point ranged from 4.69 to 10.45, and the molecular weight ranged from 20.44 to 119.84 KDa (Table 1).

**Table 1:** Identification of stress-inducible WRKY genes in Pak-choi

**Gene name**	**Accession number**	**CDS (bp)**	**Size (aa)**	**Mass (KDa)**	**pI**	**Group**	**Atortholog**	**NLS locaton**	**NucPred score**	**Nuclear localization**
*BcWRKY1*	KF430025	858	285	31.75	7.64	IIa	AtWRKY18	95-98	0.85	Nucl: 13.0
*BcWRKY2*	KF430026	2067	860	93.24	7.53	I	AtWRKY2	152-170	0.84	Nucl: 14.0
*BcWRKY3*	KF430027	1428	475	51.92	7.94	I	AtWRKY3		0.86	Nucl: 13.0
*BcWRKY4*	KF430028	1488	495	53.79	8.01	I	AtWRKY4	339-351	0.89	Nucl: 14.0
*BcWRKY6*	KF430029	1662	550	60.34	5.94	IIb	AtWRKY6	267-268	0.63	Nucl: 13.0
*BcWRKY7*	KF430030	1047	348	38.02	10.42	IId	AtWRKY7	253-273	0.82	Nucl: 12.0
*BcWRKY8*	KF430031	966	379	42.58	7.14	IIc	AtWRKY8	189-226	0.72	Nucl: 13.0
*BcWRKY9*	KF430032	987	328	37.38	5.55	IIb	AtWRKY9	113-125	0.75	Nucl: 12.5
*BcWRKY11*	KF430033	999	332	36.37	10.18	IId	AtWRKY11	226-249	0.92	Nucl: 14.0
*BcWRKY12*	KF430034	657	218	24.62	8.73	IIc	AtWRKY12	112-133	0.49	Nucl: 4.0
*BcWRKY13*	KF430035	882	293	32.97	9.36	IIc	AtWRKY13	169-201	0.74	Nucl: 13.0
*BcWRKY15*	KF430036	960	319	34.71	10.40	IId	AtWRKY15	217-237	0.86	Nucl: 11.0
*BcWRKY18*	KF430037	969	317	35.69	8.32	IIa	AtWRKY18	25-32	0.94	Nucl: 13.0
*BcWRKY20*	KF430038	1608	535	58.64	6.96	I	AtWRKY20		0.89	Nucl: 14.0
*BcWRKY21*	KF430039	1020	339	38.04	10.28	IId	AtWRKY21	247-267	0.95	Nucl: 14.0
*BcWRKY22*	KF430040	897	298	32.34	7.16	IIe	AtWRKY22	101-121	0.66	Nucl: 13.0
*BcWRKY23*	KF430041	972	323	36.10	6.91	IIc	AtWRKY23	133-152	0.74	Nucl: 13.0
*BcWRKY24*	KF430042	531	176	20.44	8.62	IIc	AtWRKY24	67-87	0.5	Nucl: 6.0
*BcWRKY25*	KF430043	1122	373	42.06	6.68	I	AtWRKY25	206-223	0.58	Nucl: 13.0
*BcWRKY26*	KF430044	894	319	35.94	9.62	I	AtWRKY26	217-230	0.64	Nucl: 12.5
*BcWRKY28*	KF430045	939	312	34.97	6.72	IIc	AtWRKY28	116-156	0.69	Nucl: 13.0
*BcWRKY29*	KF430046	927	308	34.17	8.43	IIe	AtWRKY29	108-131	0.55	Nucl: 13.0
*BcWRKY30*	KF430047	942	313	35.57	6.80	III	AtWRKY30	97-104	0.52	Nucl: 11.5
*BcWRKY31*	KF430048	1560	519	57.15	6.95	IIb	AtWRKY42	212-217	0.69	Nucl: 14.0
*BcWRKY32*	KF430049	1899	459	49.58	6.97	I	AtWRKY26	300-311	0.61	Nucl: 11.0
*BcWRKY33*	KF430050	1557	518	56.63	7.92	I	AtWRKY33		0.77	Nucl: 13.0
*BcWRKY34*	KF430051	1650	549	60.18	6.47	I	AtWRKY34	19-28	0.78	Nucl: 14.0
*BcWRKY36*	KF430052	1152	383	42.99	7.69	IIb	AtWRKY36	96-129	0.74	Nucl: 12.0
*BcWRKY39*	KF430053	1029	342	38.00	10.02	IId	AtWRKY74	247-271	0.9	Nucl: 11.0,
*BcWRKY40*	KF430054	873	290	32.43	7.82	IIa	AtWRKY40	75-89	0.72	Nucl: 10.0
*BcWRKY42*	KF430055	1551	519	57.15	6.95	IIb	AtWRKY42	212-217	0.69	Nucl: 14.0
*BcWRKY44*	KF430056	1230	409	44.89	9.19	I	AtWRKY44	360-364	0.67	Nucl: 8.0
*BcWRKY46*	KF430057	858	283	32.20	5.82	III	AtWRKY46	60-90	0.73	Nucl: 10.0
*BcWRKY47*	KF430058	1512	490	53.94	6.79	IIb	AtWRKY47		0.77	Nucl: 13.0
*BcWRKY48*	KF430059	1200	399	44.46	6.77	IIc	AtWRKY48	185-212	0.69	Nucl: 13.0
*BcWRKY51*	KF430060	597	198	22.29	7.54	IIc	AtWRKY51	112-129	0.49	Nucl: 12.0
*BcWRKY53*	KF430061	972	323	36.19	6.38	III	AtWRKY53	127-149	0.81	Nucl: 14.0
*BcWRKY54*	KF430062	1020	297	33.11	4.76	III	AtWRKY54	107-131	0.8	Nucl: 12.0
*BcWRKY56*	KF430063	564	186	21.03	7.23	IIc	AtWRKY56	79-89	0.37	Nucl: 9.0
*BcWRKY57*	KF430064	885	294	32.56	6.89	IIc	AtWRKY57	116-141	0.67	Nucl: 13.0
*BcWRKY58*	KF430065	3195	1082	119.84	9.63	I	AtWRKY58	865-913	0.96	Nucl: 14.0
*BcWRKY59*	KF430066	546	196	22.64	6.95	IIc	AtWRKY59	75-97	0.84	Nucl: 5.0
*BcWRKY60*	KF430067	927	308	34.71	8.22	IIa	AtWRKY18	103-109	0.91	Nucl: 13.0
*BcWRKY61*	KF430068	1662	398	44.24	6.44	IIb	AtWRKY61		0.66	Nucl: 12.0
*BcWRKY62*	KF430069	837	233	26.92	6.27	III	AtWRKY62	57-73	0.61	Nucl: 13.0
*BcWRKY64*	KF430070	777	258	29.36	4.82	III	AtWRKY67	108-117	0.67	Nucl: 11.0
*BcWRKY65*	KF430071	786	261	29.25	5.27	IIe	AtWRKY65	133-150	0.55	Nucl: 13.0
*BcWRKY66*	KF430072	738	452	52.12	6.96	III	AtWRKY64	121-138	0.56	Nucl: 8.0
*BcWRKY67*	KF430073	831	276	31.06	6.79	III	AtWRKY70	92-118	0.81	Nucl: 11.0
*BcWRKY68*	KF430074	960	319	36.04	6.61	IIc	AtWRKY23	116-145	0.82	Nucl: 13.0
*BcWRKY69*	KF430075	849	282	31.27	4.69	IIe	AtWRKY69	32-41	0.46	Nucl: 13.0
*BcWRKY70*	KF430076	852	283	32.01	6.23	III	AtWRKY70	92-117	0.77	Nucl: 12.0
*BcWRKY71*	KF430077	1047	277	31.53	8.45	IIc	AtWRKY71	99-125	0.46	Nucl: 11.0
*BcWRKY72*	KF430078	2529	551	59.68	6.36	IIb	AtWRKY72		0.58	Nucl: 14.0
*BcWRKY74*	KF430079	969	322	35.29	10.45	IId	AtWRKY11	211-244	0.96	Nucl: 13.0
*BcWRKY75*	KF430080	1062	353	39.33	6.85	IIc	AtWRKY75	143-167	0.73	Nucl: 13.0

*Abbreviations*: bp, base pair; aa, amino acids; CDS, coding sequence; pI, Isoelectric point; WD, WRKY domain; NLS, Nuclear location signal. NLS location indicated the distribution of the NLSs on the BcWRKY proteins.

### Phylogenetic analysis and identification of conserved motifs

**Figure 1:**
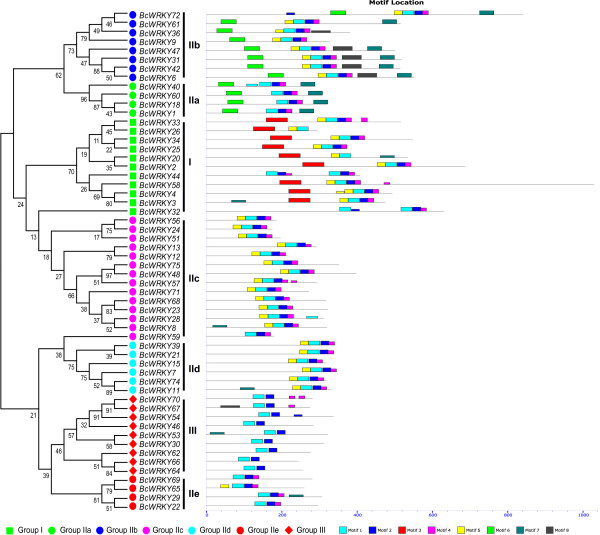
**Structural and phylogenetic analysis of putative BcWRKY proteins.** The unrooted phylogenetic tree resulting from the full-length amino acid alignment of all the BcWRKY proteins in Pak-choi is shown on the left side of the figure. The different colored balls at the bottom of the figure indicate different groups. The distribution of conserved motifs among the putative BcWRKY proteins are shown on the right side of the figure. Different motif types are represented by different color blocks as indicated at the bottom of the figure. The same color in different proteins indicates the same group or motif.

To investigate the phylogenetic relationship and structural features of the WRKY proteins in Pak-choi, an unrooted maximum likelihood (ML) phylogenetic tree and a linear distribution map of the conserved motifs in the putative BcWRKY proteins were produced (Figure 1). These were based on the multiple sequence alignment of the 56 putative BcWRKY proteins, ranging in size from 176 to 1,064 aa, using ClustalW in MEGA 5 software (using the ML method and a bootstrap value of 1000) and the MEME suite to detect conserved motifs in the BcWRKY protein sequences. The BcWRKYs were organized into three large clades, containing WRKY members that had the same or similar conserved motif distributions. The three clades were named I, II (IIa-e), and III, based on the number of WRKY domains and the type of zinc finger in the C-terminal WRKY domain. Group I consisted of 11 members containing two WRKY domains, while groups II and III contained 36 and nine members, respectively, and each member contained only one WRKY domain (Table 1). The distributions of different motifs formed groups, and the shared motifs appeared in all groups, such as motif 1, motif 3, and motif 5 (Figure 1). Of the eight motifs, motifs 1 and 3, which represented the distribution of C-or N-terminal WRKY domains, respectively, were both contained in the WRKY aa residues, and motif 5 contained nuclear localization signal (NLS) sequences, which could represent the NLS distribution of BcWRKY proteins (Table 2). Group I shared conserved motifs 1, 3, and 5, except for BcWRKY32, which lacked motif 3. Group II contained five subgroups (IIa-e) based on zinc finger types and consisted of 36 WRKY members, which contained several common and specific motifs, such as motifs 1, 2, and 4 that were shared by group II, and motifs 6 and 7 that were only found in group IIa and IIb (Figure 1).

**Table 2:** Motif sequences of 56 BcWRKY proteins identified by MEME tools

**Motif**	**Consensus sequence**	**Known motif description**
Motif1	LDDGYRWRKYGQKPVKGSPYPRSYYRCTT	WRKY
Motif2	CPVRKQVERSAEDPSIVITTY	
Motif3	NDGYQWRKYGQKVAKGNPCPRAYYRCTMA	WRKY
Motif4	EGKHNHPLPxARxSxASSTSA	
Motif5	KKSEKKVREPRVAVQTRSDVD	Nuclear location signal
Motif6	LREELNRVNEENKKLKEMLSQVxENYNSLQMHLEKLMRQQ	
Motif7	MQEVLVEQMASALTADPNFTAALAAAISSIIGGQNNT	
Motif8	KFTQGCKATKQVQKIENDPSLFRITYIGKHTCNV	

Significant motifs (e-value < 1e-100) of more than 10 amino acid length were predicted by MEME search.

### Alignment and comparison of WRKY domains

**Figure 2:**
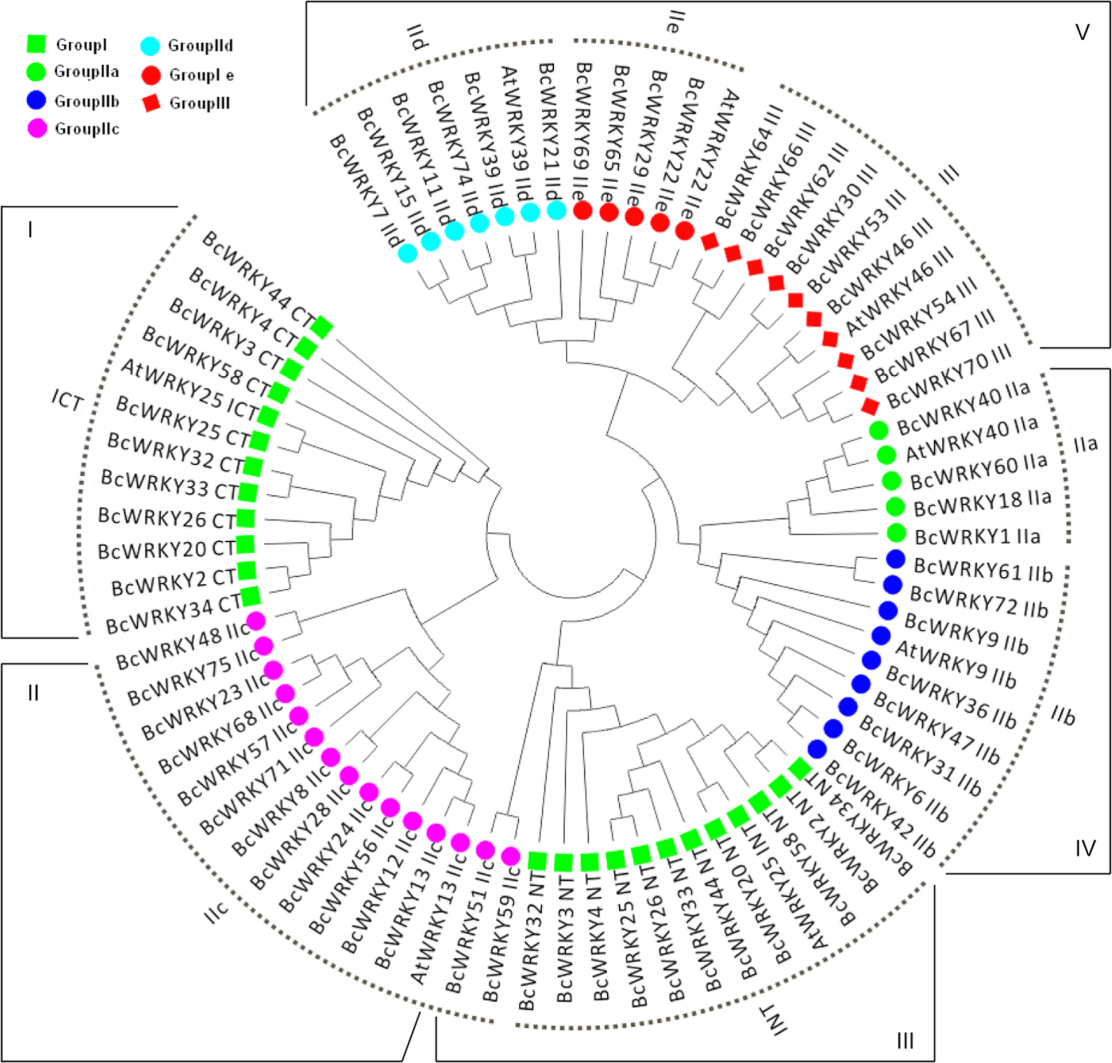
**Phylogenetic analysis of the WRKY domain.** The amino acid sequences of the WRKY domains in Pak-choi and *Arabidopsis* were aligned and used to construct a maximum likelihood method phylogenetic tree using MEGA 5. The different colors balls at the bottom of the figure indicate different groups.

To compare phylogenetic relationships among the WRKY domains, 67 WRKY domains that contained the highly conserved N-terminal WRKYGQK motif and C-terminal zinc finger were extracted and aligned from 56 BcWRKY proteins. The WRKY domains were classified into the eight subgroups and named ICT, INT, IIa-e, and III (Figure 2 and Additional file 2: Figure S1). Eleven members of group I, which contained two WRKY domains, including a C-or N-terminal WRKY domain, were separately divided into groups INT and ICT. The 36 WRKY domains of group II each contained one WRKYGQK motif and a C_2_C_2_-type zinc finger motif (C-X_5_-C-X_23_-H-X_1_-H), and could be classified into five distinct subgroups (IIa-e) based on different conserved motifs contained in the WRKY domain. Group IIa was comprised of *BcWRKY1*, *BcWRKY18*, *BcWRKY40*, and *BcWRKY60*, which each contained motifs 1, 2, and 4 in the WRKY domain and motifs 6 and 7 outside of the WRKY domain. Group IIb had nine members that each contained motifs 1, 2, 4, and 5 in the WRKY domain and motifs 6 and 8 outside of the WRKY domain. Group IIc and IId had 14 and eight members, respectively, and contained motifs 1, 2, 4, and 5 in the WRKY domains, but the motif positions were different. The conserved motifs of group IId only occurred at the C-terminus of the WRKY proteins. The group IIe WRKY domains only contained motifs 1, 2, and 4 (Figure 1). Group III had nine WRKY members that only shared motifs 1 and 2, and whose WRKY domains contained the C2HC-type (C-X7-C-X23-H-X1-C) of zinc finger motif (Additional file 2: Figure S1). The patterns of WRKY domains and zinc finger motifs in the BcWRKY proteins were similar to the patterns of *Arabidopsis* WRKY domains (Additional file 2: Figure S1) and were consistent with a previous study on groups II (C-X_4-5_-C-X_22-23_-H-X_1_-H) and III (C-X_7_-C-X_23_-H-X_1_-C) [4].

By comparing the 67 WRKY domains, a phylogenetic tree with five clades was constructed (Figure 2). In terms of the eight WRKY domain patterns, the ICT group was placed in clade I, and the next branch, clade II, consisted of group IIc. This was considered as an intermediate between clades I and III, which involved groups ICT and INT. While clade III, including the INT group, was placed as an original node, the remaining 31 domains were clustered into clade IV (groups IIa and IIb) and clade V (IId, IIe, and III). These results illustrated the evolution of Pak-choi WRKY domains from group I to group II or III (Figure 2). For example, BcWRKY51 and BcWRKY59, belonged to group IIc, but they were clustered into the INT group in the phylogenetic tree (Figure 2), and BcWRKY32 protein belonged to group I, containing two WRKY domains, but clustered into the IIc group in the phylogenetic tree (Figure 1). A similar evolutionary pattern for WRKY domains has been reported in *Arabidopsis* and rice [8].

### Subcellular localization analysis of BcWRKYs

**Figure 3:**
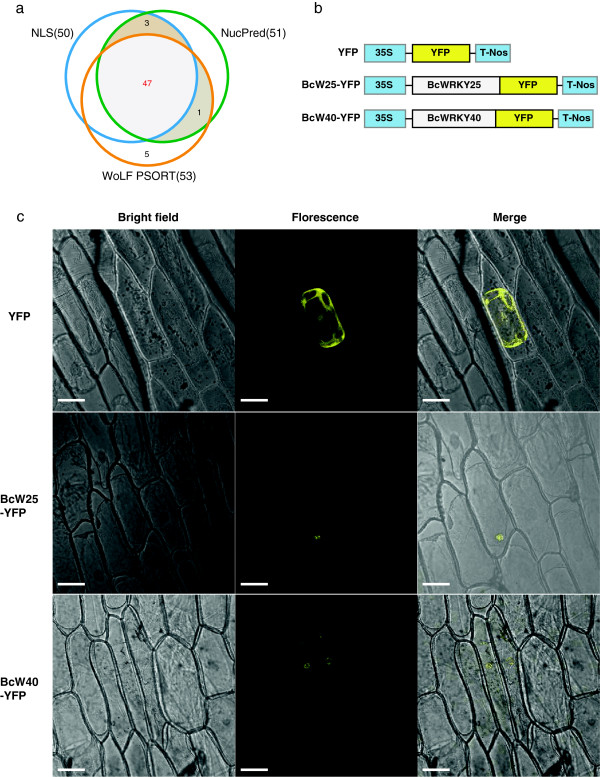
**Subcellular localization analysis of BcWRKYs.** The subcellular localization analysis of the putative BcWRKY proteins used NLStradamus, NucPred, and WOLF PSORT with the default settings to detect nuclear localization scores. **(A)** two yellow fluorescent protein (YFP) marker expressing *BcWRKY* fusion genes, BcWRKY25-YFP and BcWRKY40-YFP, were constructed; **(B)** BcWRKY25-YFP and BcWRKY40-YFP were introduced into onion epidermal cells by particle bombardment with the YFP signal as an indicating marker to test the subcellular localization of BcWRKY proteins; **(C)** the upper panel, the corresponding bright field, fluorescence, merged fluorescence image of YFP control; the middle panel, the corresponding bright field, fluorescence, merged fluorescence image of BcW25-YFP; the lower panel, the corresponding bright field, fluorescence, merged fluorescence image of BcW40-YFP. Scale bars: 20 μm.

To investigate the subcellular localization of putative BcWRKY proteins, we used NLStradamus with the default settings. We found 50 BcWRKY proteins contained NLSs. Additionally, we used NucPred and WOLF PSORT to predict the nuclear localization scores of the BcWRKY proteins. Fifty BcWRKY proteins had a NucPres-score of ≥ 0.5 and 53 had nuclear localization scores of ≥ 7 (KNN = 14) using WOLF PSORT (Table 1). A consensus of the results generated predicted that most BcWRKYs (47/56) localized at the nucleus (Figure 3A). Additionally, we used a transient expression system in onion epidermal cells to test the subcellular localization of BcWRKY proteins. The yellow fluorescent marker protein (YFP) was fused to BcWRKY25 and BcWRKY40 and the expression of the fusion genes was tracked by the marker’s signal (Figure 3B). When YFP alone was expressed the fluorescence was observed in the cytosol and nucleus (Figure 3C, upper panel), while the yellow fluorescence of the BcWRKY25-YFP and BcWRKY40-YFP fusions were observed in the nuclear region (Figure 3C, middle and lower panel, respectively). Thus, BcWRKY25 and 40 were localized to the nucleus, which agreed with the protein subcellular localization prediction. These results indicate that the properties of the BcWRKY proteins define them as transcription factors.

### Expression patterns of BcWRKYs under multiple abiotic stresses

**Figure 4:**
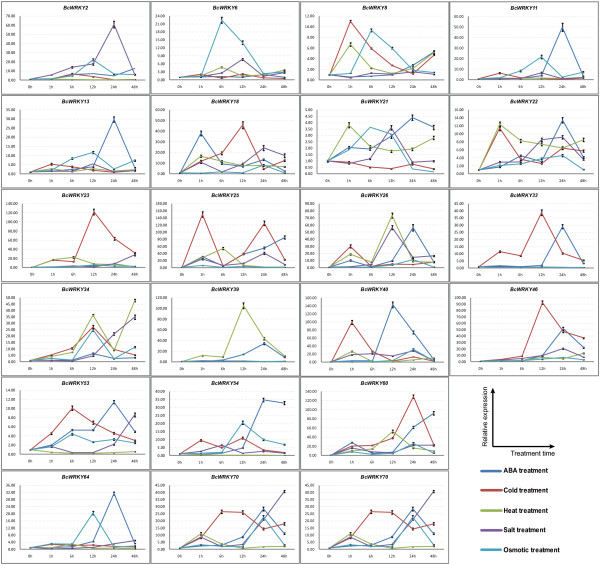
**Expression patterns of the 22 *BcWRKY*s under abiotic stresses.** Pak-choi plants were subjected to ABA, cold, heat, high salinity, and osmotic treatments, respectively. The 22 stress-inducible *BcWRKY*s were significantly expressed in response to multiple abiotic stresses in the leaves and their transcript levels were quantified against *BcGAPDH* transcript levels using 2^–ΔΔCT^, ΔΔCT = ΔCT (treated sample) − ΔCT (untreated sample), ΔCT = CT_target_ − CT_*BcGAPDH*_.

Among the 56 isolated WRKY genes, 22 genes were detected and found to be significantly induced in response to ABA and abiotic stresses in Pak-choi leaves. Of the 22 expressed stress-inducible *BcWRKYs*, 19 were up-regulated in at least one of the five treatments (ABA, salinity, cold, heat, and osmosis) and six genes were down-regulated under heat treatment (Figure 4). During multiple abiotic stress treatments, *BcWRKY*25, *40*, *60*, and *75* were all highly expressed during an ABA treatment time course. Similarly, *BcWRKY*25, *26*, *34*, *39*, and *60* under cold stress treatments, had significantly upgraded expression levels. A heat treatment also strongly induced high expression levels in some genes, such as *BcWRKY25*, *26*, *34*, *39*, and *60* (Figure 4). Additionally, *BcWRKY2*, *6*, *23*, *26*, *34*, *40*, *60* and *70* had high expression levels under salt treatment and osmotic treatment. Meanwhile, most *BcWRKY*s’ expression levels peaked at the 12-h or 24-h time-points. However, the expression peaks for *BcWRKY22*, *25*, *26*, and *40* occurred at the 1-h time-point and *BcWRKY53* and *BcWRKY70* peaked at the 6-h time-point under cold treatment (Figure 4). Interestingly, *BcWRKY18*, *25*, *40*, *60*, *70* were all predominantly expressed in response to multiple stress treatments, and the simultaneous expression of the *BcWRKY*s was detected. These expression processes exhibited some low to high or high to low curve changes over the 48 h time course, showing that the inducible responses of *BcWRKY*s to multiple abiotic stresses is a dynamic process. The results indicated that stress-inducible *BcWRKY*s were strongly induced and coordinately mediated in response to multiple abiotic stresses in Pak-choi leaves.

### Co-regulatory networks of BcWRKYs in response to multiple abiotic stresses

**Figure 5:**
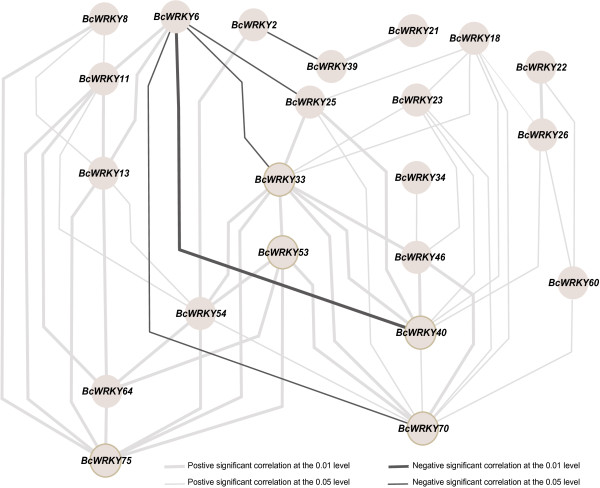
**Co-regulatory networks of the 22 stress-inducible *BcWRKY *genes.** The co-regulatory networks of stress-inducible *BcWRKY*s were established based on the Pearson correlation coefficients of stress-inducible *BcWRKY* gene pairs using log_2_ transformed qPCR data, which involved 22 nodes and 56 regulatory edges. All of the Pearson correlation coefficients of co-regulatory gene pairs were significant at the 0.05 or 0.001 significance level (p-value), and the different edge line styles indicate the different significance levels of the co-regulated gene pairs.

Co-regulatory networks were established based on the Pearson correlation coefficient of stress-inducible *BcWRKY* gene pairs using log_2_ transformed qPCR data (Figure 5). All Pearson correlations that were significant at the 0.05 significance level (p-value) were collected and visualized by Cytoscape 2.8 to construct stress co-regulatory networks of *BcWRKY*s. There were 22 nodes representing 22 stress-inducible *BcWRKY*s separately connected by 56 edges, which represented the Pearson correlation coefficients of the co-regulatory gene pairs (Figure 5). In the co-regulatory networks, most co-regulatory gene pairs (51/56) appeared to have positive significant correlations, except for four *BcWRKY* gene pairs, *BcWRKY2-BcWRKY39*, *BcWRKY6-BcWRKY70*, *BcWRKY6-BcWRKY25*, and *BcWRKY6-BcWRKY33*, which had negative correlations (0.05). In addition, *BcWRKY6-BcWRKY40* had a large negative correlation at the 0.001 significance level. Among 51 positively correlated gene pairs, 27 and 29 gene pairs were significant at the 0.05 and 0.001 levels, respectively (Figure 5). Among the 22 mutually linked nodes, *BrWTKY33*, *40*, *54*, and *70* had more edges, with 10, 8, 7, and 10, respectively (Figure 5). These results suggested that they represented central nodes in the co-regulatory networks of *BcWRKYs* in response to multiple abiotic stresses. All data used to calculate the correlations are shown in Additional file 3: Table S3.

To validate the co-regulatory relationships of the *BcWRKY* genes, we constructed an evident interaction co-regulatory network of WRKY orthologs through the STRING 9.1 database based on the stress-inducible *BcWRKY* orthologs in *Arabidopsis* (Additional file 4: Figure S2). Among the 11 co-regulatory WRKY members, each of them existed in at least two mutual relationships with others in the given datasets (Databases, Text-mining, Homology, Experiments, Neighborhood, Co-occurrence, and Co-expression). Those were further verified, and they supported the co-regulatory networks of stress-inducible *BcWRKY*s in which *WRKY33*, *40*, *53* and *70* represented central nodes.

## Discussion

This study reported the isolation and identification of 56 stress-inducible *BcWRKY* genes (Table 1) using a homologous gene cloning method from a stress-induced Pak-choi cDNA library. We also systematically surveyed the structure, phylogeny, and conserved motifs of the BcWRKYs and measured the putative protein localization. In addition, a co-expression analysis of *BcWRKY*s was performed to explore the co-regulatory information of WRKY TFs. Based on a sequence alignment of the BcWRKY domains (Additional file 2: Figure S1), we clearly found phenomena similar to those previously reported [5]. This indicated that possessing different numbers of WRKY domains and zinc finger motifs, and structural variations of the WRKY domain, may produce some novel WRKY members (Additional file 2: Figure S1). For example, the BcWRKY32 protein contains two of the same type of WRKY domains, belonging to Group I; however, in a phylogenetic analysis it can be clustered together with Group IIc members, while BcWRKY59 with WRKY domain features of Group IIc was clustered into a clade with Group IId members because of the absence of some motifs (Figure 1). These results also support the belief that group I WRKYs may represent the ancestral form of the WRKY family [33]. Additionally, the conservation of WRKY domains implies a conserved function for WRKY TFs [34]. For example, the subcellular localization of BcWRKY25 and BcWRKY40 proteins was in the nucleus, which was appropriate for TFs, thus supporting their classification as functional TFs [35].

Although the mechanisms of the WRKYs’ responses to multiple abiotic stresses needs further investigation, the co-expression analysis, which has the ability to measure large numbers of gene expressions, provides a powerful tool for identifying groups of genes and discovering novel regulators involved in the signal transport of plant stress responses. Previous studies have performed co-expression analyses [29,30,36], and found many unknown relationships and novel genes encoding proteins involved in similar expression patterns under different conditions. The co-expression analysis of *Arabidopsis* and Pak-choi WRKY orthologs (Figure 5 and Additional file 4: Figure S2) indicated co-regulatory relationships and key regulators of 22 stress-inducible *BcWRKY*s in response to multiple abiotic stresses, and it further indicated the important roles of the WRKY TF mutual interactions to mediate complex biological processes [37]. These findings will help in identifying and understanding more interactive relationships among the WRKYs and will elucidate more co-regulatory relationships for WRKYs under multiple abiotic stresses.

## Conclusions

This study isolated and characterized 56 Pak-choi stress-inducible WRKY genes, indicated that 22 *BcWRKY* genes co-regulated multiple abiotic stress responses in Pak-choi leaves, and established a co-regulatory network of stress-inducible *BcWRKY*s. The co-regulatory network showed that *WRKY33*, *40*, *53* and *70* were central regulators and had potentially interactive relationships. This study also revealed a method of measuring the common and distinct functions of stress-inducible *BcWRKY*s among multiple abiotic stress responses, which may aid in exploring the molecular mechanisms of WRKYs in response to abiotic stresses in plants.

## Methods

### Plant materials, growth conditions and stress treatments

Pak-choi (*B. campestris* ssp. *chinensis* cv. *suzhonqing*) was used for all experiments. Seedlings were soaked in distilled water for 0.5 h, and then germinated in plastic Petri dishes containing filter paper saturated with distilled water in darkness at 22°C for 2 days. Seedlings were then transferred to 4 L hydroponic containers containing continuously aerated 1/2 Murashige and Skoog (MS) liquid solution (pH 5.8, without agar and sugar). The 1/2 MS liquid solution was changed once every 3 days. Three-week-old seedlings were transferred to new 1/2 MS liquid solution (pH 5.8, without agar and sugar) for multiple stress treatments under a continuous time course (0, 1, 6, 12, 24, and 48 h). For ABA, salt and osmotic treatments, seedlings were exposed to 1/2 MS solution (pH 5.8) containing 100 μM ABA, 200 mM NaCl and 15% (w/v) polyethylene glycol (PEG), respectively. For cold and heat treatments, seedlings were exposed to the 4 and 38°C conditions in 1/2 MS solution (pH 5.8), respectively. All seedlings were placed under the same growth conditions, except for the different treatment factors, and exposed to 1/2 MS solution at 22°C as controls. The seedlings were harvested under a continuous time courses (0, 1, 6, 12, 24, and 48 h) in three biological replicates for RNA preparation.

### Cloning and identification of the BcWRKY members in Pak-choi

Total RNA was extracted from Pak-choi roots, stems, and leaves under multiple abiotic stress conditions using the RNAeasy mini kit (Tiangen, Beijing, China). A mixture of total RNA (1 μg) was used for first-strand cDNA synthesis using a superscript II kit (Takara, Dalian, China) following the manufacturer’s instructions to construct a stress-induced Pak-choi cDNA library. To clone *BcWRKY* genes, we first designed degenerate primers (5′-YTTYTGNCCRTAYTTNCKCCA-3′, Y = C/T, R = A/G, K = G/T, N = A/G/C/T) and 5′-Oligo(dT)20MN-3′(M = A/G/C, N = A/G/C/T) to amplify the conserved regions of the WRKY orthologs based on sequence information from the *A. thaliana* WRKY gene family in TAIR10 (http://arabidopsis.org/index.jsp) and the Chinese cabbage *chiifu* genome in BRAD (http://brassicadb.org/brad/). Based on the results of sequenced polymerase chain reaction (PCR) products from the conserved region of each of the WRKY orthologs and the full length sequences of Chinese cabbage WRKY orthologs (data not shown), we designed gene-specific primers (Additional file 1: Table S1) and performed 5′-and 3′-RACE (Smart RACE cDNA amplification kit; Clontech, Mountain View, CA) to amplify the full-length cDNA sequences of *BcWRKY*s in the stress-induced Pak-choi cDNA library. The 56 open reading frames (ORFs) from the stress-inducible *BcWRKY* cDNA sequences were amplified by reverse transcription-polymerase chain reaction (RT-PCR) using gene-specific primers (Additional file 1: Table S1). PCR reactions included a pre-incubation at 94°C for 5 min, followed by 30 cycles of denaturation at 94°C for 30 s, annealing at 55–65°C for 2 min, and extension at 72°C for 10 min. The amplification fragments were cloned in to PMD-19T (Takara). The gene ORF size was confirmed and sequenced using the ABI3730 sequencer (Applied Biosystems, Foster City, CA).

### Multiple sequences alignments and phylogenetic analyses

The amino acid sequence alignments of putative *BcWRKY*s were performed using ClustalW implemented in the MEGA 5 software [38] with the default settings. They were visualized and manually modified using Jalview 2.7 [39]. Phylogenetic trees of BcWRKY proteins and WRKY domains were built using the maximum likelihood method in MEGA 5. The confidence level of the monophyletic group was estimated using a bootstrap analysis of 1000 replicates.

### Identification of conserved motifs

The 56 putative BcWRKY protein sequences used for the phylogenetic analysis were detected by MEME [40] to analyze possible conserved motifs using the default parameters, except that the maximum number of motifs to identify was defined as eight and the maximum width was set to 200.

### Nuclear localization analysis

A subcellular localization analysis of deduced BcWRKY proteins was performed by bioinformatics predictions and experimental approaches. NLSs were detected using NLStradamus [41], and nuclear protein scores were calculated separately using WOLF PSORT [42] and NucPred [43] with the default settings. Meanwhile, two expression vectors were constructed (Figure 3B) to investigate the subcellular localizations of BcWRKY TFs using a transient expression system in onion epidermal cells. The full-length coding sequences of *BcWRKY25* and *BcWRKY40* were amplified using Gateway-specific primers (Additional file 5: Table S2) cloned into an entry vector, and then subcloned into pEarleyGate101 by Gateway technology (Invitrogen, Carlsbad, CA). The yellow fluorescent marker protein (YFP) was fused to BcWRKY25 and BcWRKY40. Gold particles with a diameter of 1 μm coated with 35S:BcW25-YFP, 35S:BcW40-YFP, and 35S:YFP (Figure 3B) were introduced into onion epidermal cells using particle bombardment (PDS-100/He particle delivery system; Bio-Rad, Hercules, CA). After incubation at 22°C for at least 12 h under darkness, fluorescence and bright-light images were observed by laser scanning confocal microscopy (Leica, TCS SP2, Wetzlar, Germany).

### RNA isolation and quantitative real-time PCR (qPCR)

Leaf samples were obtained from control and multiple abiotic stress-treated plants for total RNA extraction with an RNA kit (RNAsimply total RNA Kit, Tiangen). Total RNA was treated with DNase I (Takara) for potential genomic DNA contamination. For qPCR analysis, 1 μg of total RNA was used to synthesize the first-strand cDNA using the PrimeScript™ RT reagent Kit (Takara) for RT-PCR in a 20-μl reaction volume according to the manufacturer’s instructions. The cDNA reaction mixture was diluted 1:10 with EASY Dilution for Real Time PCR (Takara), and 2 μl was used as the template in the 20-μl PCR reactions. PCR reactions included a pre-incubation at 95°C for 4 min, followed by 40 cycles of denaturation at 95°C for 30 s, annealing at 58°C for 30 s, and extension at 72°C for 30 s. All the reactions were performed in a 7500 Fast Real-Time PCR System (Applied Biosystems) using SYBR® Premix Ex Taq (Takara). After the PCR was run, a melting curve (65–95°C, at increments of 0.5°C) was generated to confirm the specificity of the amplification. The gene glyceraldehydes-3-phosphate dehydrogenase (*BcGAPDH*) was used as an internal control. The gene-specific primers that were used to detect transcripts are listed in Additional file 1: Table S1. The relative gene expression was calculated as previously described [44]. The gene expression was measured from at least three biological replicates (three technical replicates for each biological replicate).

### Pearson correlation and co-regulatory networks

To display the regulatory relationships of stress-inducible *BcWRKY* genes that mediated multiple abiotic stresses, the Pearson correlation coefficients of stress-inducible *BcWRKY* gene pairs were calculated using a house Perl script based on log_2_ transformed qPCR data. All of the gene pairs whose Pearson coefficient was significant at the 0.05 significance level (p-value) were collected for a gene co-regulatory network analysis. Co-expression networks were graphically visualized using Cytoscape version 2.8 [45] based on Pearson correlation coefficients of *BcWRKY* gene pairs. The nodes represent genes and the edges between nodes represent gene pairs expressing correlations (hypothetical interactions). The different edge line styles indicate different correlation levels between corresponding nodes, which, in turn, indicate different interaction strengths between the co-regulated gene pairs. In addition, we compared the protein-protein relationships of stress-inducible *BcWRKY* orthologs in Arabidopsis using STRING 9 (http://string.embl.de/) with the default program parameter settings.

## Competing interests

The authors declare that they have no competing interests.

## Authors’ contributions

JT, FW, and XH conceived the project. JT, ZW, and ZH prepared the plant materials and carried out the gene cloning and expression analyses. JT and FW performed analyzed the data; AX provided advice on protein classification. JT and XH prepared the manuscript. FW, AX and XH revised and proofread the manuscript. All authors read and approved the final manuscript.

## Supplementary Material

Additional file 1: Table S1 Primers used for RT-PCR and real-time PCR. Click here for file

Additional file 2: Figure S1 Alignment of WRKY domains between Pak-choi and Arabidopsis. Click here for file

Additional file 3: Table S3 Pearson correlation coefficient of stress-induced *BcWRKY*s. Click here for file

Additional file 4: Figure S2 A confidence co-regulatory network of WRKY orthologs in Arabidopsis. Click here for file

Additional file 5: Table S2 Gatway cloning primers for subcelluar localization. Click here for file
