# Supplementary material for: Characterization and co-expression analysis of WRKY orthologs involved in responses to multiple abiotic stresses in Pak-choi (Brassica campestris ssp. chinensis)
Source: BMC Plant Biol. 2013 Nov 25;13:188. doi: 10.1186/1471-2229-13-188 (PMC4222839; doi:10.1186/1471-2229-13-188)
Supplement: Additional file 1: Table S1 — Primers used for RT-PCR and real-time PCR. [file 1471-2229-13-188-S1.pdf]

Supplemental Table S1 Primers used for RT-PCR and real-time PCR

Gene name	Usage of primer	Forward Primer (5'-3')	Reverse Primer(5'-3')	Amplicon Lenght (bp
BcWRKY1	ORF amplication	ATGGACTGTTCTTCCTTTCTTGAC	TCATGTTCTAGACTGCTCCATTAAC	858
	qRT-PCR	GGATCTGGTTCATAGTGGTCAG	TAACGAAGACGCCATTTGCCGAAC	125
BcWRKY2	ORF amplication	ATGTCTGGTTTTGATGAAAACGT	TCAAATCTGTGGTAATCTACTC	2067
	qRT-PCR	CGGTTACTCGTTCGGTTTAGG	CGGTTGAGTCATATACGGGTG	112
BcWRKY3	ORF amplication	ATGGCAGAGAATGAAGAAGAACAAC	TCAAGTGATTTGCTCTTCTTTAAGC	1428
	qRT-PCR	AGAAGCAAGTCAAAGGTAGCG	GATTGTGCTGACCCTTGTAAATG	135
BcWRKY4	ORF amplication	ATGTCGGAGGAAAAAGAAGAACT	TTAAATGATTTGCTCTTCTTTCAG	1488
	qRT-PCR	CTGAAGACGAGCCTGATG	TTACGCCACCTGTATCCA	172
BcWRKY6	ORF amplication	ATGACGCAGGATCTGACAGGTTG	CTATTGATTTTTGTTGTTTCCTTC	1662
	qRT-PCR	CGGAAGATAGGACTCGTTCAG	TTGTTAGACTCGGTTTCAGGAC	112
BcWRKY7	ORF amplication	ATGACGGTGGAGCTAATCATGAG	TTAAAGAGTTTTTTCATGAGGC	1047
	qRT-PCR	CGGTGGATCTGCGTTTAGAG	GGTTCTTGATGTGGTTTTCCG	141
BcWRKY8	ORF amplication	ATGTCTAATGAAACCAAAGATC	TCAAGGTTCTTGTTTGAAGA	966
	qRT-PCR	ACCAAGATCCAACAGTCGTG	AGCCTGGAGATAGAGACGATG	132
BcWRKY9	ORF amplication	ATGGATCTTTCTCTCAGACTTGAGAAAG	CTACATCATCCAATTCAACCGGTTTTGG	987
	qRT-PCR	AGCTCGAGATGAAATTCCCG	AGTGAAAGCCCTAATTCCCC	145
BcWRKY11	ORF amplication	ATGGCCGTCGATCTTATGCGCT	TCATGCCGAAGCAAACACCA	999
	qRT-PCR	GACTTATGAAGGAGAGCACCG	AACACCAAATCATTAACGCCTG	81
BcWRKY12	ORF amplication	ATGGAAGGAGGAAGAAGAGTAG	TTAAAAGGAAGAGAGACAATC	657
	qRT-PCR	TGACGATGGATACAAATGGCG	ACCATTCTACAATCTTCCGACAG	141
BcWRKY13	ORF amplication	ATGGGTACGATAAACCAAGGAAT	TCACCAGAAGAAGTTGGAGAG	882
	qRT-PCR	GAAGCCGTTAGGAGTAGTG	GGATGTTGAGTGTTCTTGAC	168
BcWRKY15	ORF amplication	ATGGCGGTGGAGCTCATGACTAG	TCAAGACGATTCCAAAATGAG	960
	qRT-PCR	AGTCCGAGCACATGAAACAC	ACTATTGTGGAGAGGCTTTGAG	142
BcWRKY18	ORF amplication	ATGGACTGTTCTTCGTTTCTCG	TCATGTTCTAGACTGCTCCATC	969
	qRT-PCR	CGAAGGAGGAGGTCAAGGTT	TGGTGATGTAGTGCGTAGTAGT	157
BcWRKY20	ORF amplication	TCATGTTCTAGACTGCTCCATC	CTAAGGACCCGACTGTATTCTC	1608
	qRT-PCR	GGCGTAAGTATGGTCAGAAGG	CATCGTGATTGTGTTTGCTGG	98
BcWRKY21	ORF amplication	ATGGAGGAGATAGAAGGAAC	CTCAAACCGTGACAACTTAA	1020
	qRT-PCR	CCCACTGATTCCCTCACTTTAG	TGAGCCTGTAACCGTTCATG	119
BcWRKY22	ORF amplication	CTCAAACCGTGACAACTTAA	TCATATTCCACCGCTAGCTG	897
	qRT-PCR	AGCCCTGTTTTCACCTTCG	TTCTGGTTTTGTCTCTGTCGG	149
BcWRKY23	ORF amplication	ATGGAGTTTACTAGTTTTTATC	CTACTCTTCCTTCAACATATGAC	972
	qRT-PCR	CTAACTCGTCCTCGTTTTCCTC	TCTCTTTGTCGCTTCAGGC	148

BcWRKY24	ORF amplication	ATGGAAAGACAAGACATAATTC	TCAAAGATTAGAAAAACTGGAG	531
	qRT-PCR	CCTCCGTTAGCACTTCCTG	TCTTCCCTTGATCTCCGATTTG	115
BcWRKY25	ORF amplication	ATGTCGTCTACTTCTTTCTC	TCATGACCGACGCAGCGAGGTTG	1122
	qRT-PCR	AGATTTTCCCTGGCAGCTAC	TCCAAACTCACGATGAACCG	124
BcWRKY26	ORF amplication	ATGGCCTCTTTCAACCAGCAAAG	TTATGCTTTCCTTCGTAAGTAG	894
	qRT-PCR	CTCTGAAGATGGGTACAACTGG	TGTGAGGCAGTTTGGATACG	103
BcWRKY28	ORF amplication	ATGTCCAATGAAACCAAAGAT	TCAATGCTCTTGCTTGAAGAAAATG	939
	qRT-PCR	ACCAATTCCCTTCATCCTTCTC	GAGGCAATCAGTGAAGGAGTAG	116
BcWRKY29	ORF amplication	ATGGGTGAGGTGACTTATATGGAC	TTAGTAATTCCATAAGTACCCATTG	927
	qRT-PCR	TGGACGAAGGAGATTTAGAAGC	GGCAAAACGGAGGTGAAAAC	99
BcWRKY30	ORF amplication	ATGGAGAGGAAAAGTAATAGTG	TTAGAACCCTCTAAAACCTTTGA	942
	qRT-PCR	AACAAGTCCAGAGATCCAACG	GTGTTCTTGTGGCTGGTTTG	140
BcWRKY31	ORF amplication	ATGTTTCGTTTTCCGGTGAGT	TTATTGCCTCTCAACACTGCTTGTC	1560
	qRT-PCR	CCTATCAGGTTCCACAATGTCG	GAGGTCTAGTGTAATGGTTGGG	139
BcWRKY32	ORF amplication	ATGGAGAAGAGCACGCAGGAGAC	CTAGCCACCAACAACATCCAATGC	1899
	qRT-PCR	AGGAAGATGTAAGCGTGGTTG	GATGGGTGAGTAGGAGATGTTTC	82
BcWRKY33	ORF amplication	ATGGCTGCTTCTTCTCTTCTTAC	TCAGGACAAAAACGAATCGAAAAACG	1557
	qRT-PCR	CTACTTCTGGTTCGCTTGGAG	TTCCATTACCATTCCCACCG	129
BcWRKY34	ORF amplication	ATGCCTGGTTTTGATAATAACT	TCATATTTTTGGTAGTCTACTCA	1650
	qRT-PCR	CACTGGACTATCGGGAACAAG	GCTTTGAACCTGCATTGATCC	133
BcWRKY36	ORF amplication	ATGATCAAAGAAGAGATCGATTC	TCACTGCCGTCCAAAAAGTCTG	1152
	qRT-PCR	AAACGAATCCTCTTCCACGAG	CTTTGCACCTGTTTCCTTACG	76
BcWRKY39	ORF amplication	ATGGAGGAAGTTGAAGCTGCAAAC	TCAGGTGTGAGCTGACTGGGAGGAG	1029
	qRT-PCR	TGCTATTCCCCAAGTGTTTCC	CTGCGACATATGATCAGACCC	135
BcWRKY40	ORF amplication	ATGGACCAGTACCCATCATCTTTG	CTACTTGTCGGTTTGATTCTGTTG	873
	qRT-PCR	GTGAACATAACCATCCAATGCC	CAATGGTAGTCACAGGCTCAG	111
BcWRKY42	ORF amplication	ATGTTTCGTTTTCCAGTGAGTCT	CTATTGCCTGTCACCGCTGCTC	1551
	qRT-PCR	ACCATGTTGCCTTGTTCCTC	TTGGATGCTGATTCTGTGAGG	92
BcWRKY44	ORF amplication	ATGGATGTGAAAGAGAGTGAAAG	TTAAATGGCTTGATTAGAATGTTG	1230
	qRT-PCR	TTCCTAACCTAGTCCCGAGAG	TGCTTCTGTCCGTATTTCCTC	106
BcWRKY46	ORF amplication	ATGGTGATGGAAGAGAAACTCG	TCACCAATTCAAAACGTCACAC	858
	qRT-PCR	ACTACGAGATTGAGAATGCTGG	AGTTCTCAACGGAAGCAGG	135
BcWRKY47	ORF amplication	ATGGAAGAACACTCTCAAGATGGC	TTAGGTGGTGGAGAAAGTGGTGC	1512
	qRT-PCR	TCCCATACACTTCCACAATCG	GTTGGTGAGGTCTAGAGTTATGG	74
BcWRKY48	ORF amplication	ATGGAGAAGAAGATAGAACATC	TCATTTCTTATCCTCTTCAACG	1200
	qRT-PCR	GTGGAGTGAAGAAGAGAGTGG	TCAAGGATTGGTGACGTGAG	136

BcWRKY51	ORF amplication	ATGAATCCCTCTCAAAGCCCTAGC	TCAACATGGTGGAAATGTTTGGA	597
	qRT-PCR	ACTTCATCTCCGACAACCATC	CTCTCCATCTTTACTTCCCCTATTT	123
BcWRKY53	ORF amplication	ATGGAAGGTAAAAAGGATATGT	ACCAGACTCGATTTATTATTAA	972
	qRT-PCR	CGAAGTGACGTACAGAGGAAC	GTTAGGCGTTTGAGCAATCTG	121
BcWRKY54	ORF amplication	ATGGATCCAAATAGTAACAACAC	TCATATGGCATTTGTTCCTTC	1020
	qRT-PCR	AGAGCAAGAGAACGACATCAG	GCCACGCCAATGATAAATCG	137
BcWRKY56	ORF amplication	ATGGAAGGGATAGACAACACACAC	TTACAAATCAGAAACTCTGGTG	564
	qRT-PCR	AAGGGAAAGGCAAGAGATCG	GCATCTGTAATAGCTCCTGGG	149
BcWRKY57	ORF amplication	ATGACCGATCCTGAAGATCCCG	TCAAGGATTGCGCATGGTTTGA	885
	qRT-PCR	ACTCAACCCCTAAAACACCG	TTCTGTCCATACTTACGCCATC	145
BcWRKY58	ORF amplication	ATGGCGGTGGAGGACGATGTATC	TTAACCTCTTTCTATTCTTGGCGGT	3195
	qRT-PCR	AACAAGACCCACGATCACAG	CTCCACCGCCAAATAGATACG	74
BcWRKY59	ORF amplication	ATGAACTACCCTTCAAACCCT	TCACACAGTCTGATCCGAGTTTG	546
	qRT-PCR	TCCATTTCCGAGGCATTACC	AGGGCTTGGGTGATTATGTC	130
BcWRKY60	ORF amplication	ATGGACTGTTCTTCTTCTCTCG	TCATGTTCTAGACTGCTCCATC	927
	qRT-PCR	CAAGAGATAACCCTTCGCCTAG	AAGTGGTTATGTGTCCCTTCG	136
BcWRKY61	ORF amplication	ATGGAGAAGGAAGAGTTCTTG	TTAAGGGCTCTTCTCGGCTTCACTTC	1662
	qRT-PCR	GCAACATTAGCACCCATCAAC	GATGTGGAGACGATGAGGAAG	108
BcWRKY62	ORF amplication	ATGAAGGAGTGTATGACAGAGAAC	TCATGATAAGTCACTTGATGTGCG	837
	qRT-PCR	ATCCAATAACTCATCTCCCCAAG	TTCGTGTCTGCGGTAATCTG	118
BcWRKY64	ORF amplication	ATGTTTCACGGCGCTGACAAGG	TCAAACGAGGAAAGGATTGTGAT	777
	qRT-PCR	TTCTAACACTCAACCACACCG	GTTTTGAAGCGGAGTTGGTG	139
BcWRKY65	ORF amplication	ATGAAGCGAGGTCTAGATATG	TCAAAAGATCTCAACCTGTG	786
	qRT-PCR	TCACCAAACTCTCCAACCTTC	AATCTGACGGTGGAGTTGTG	148
BcWRKY66	ORF amplication	ATGTCTCACGAAAGCGCTTACCGC	TCAAACGTGGAAAGGATTGTAAT	738
	qRT-PCR	TTGATTCCTTCTCCTTCGCTC	TGGGTTGCCTTGTTCTGAG	85
BcWRKY67	ORF amplication	ATGGATGTTGCTAATAATAAC	CTACCAACTCCAAAGATCTG	831
	qRT-PCR	GAGGAAGTATGGACAGAAGGAG	CATCTTGGATTCGGGCTCTAG	130
BcWRKY68	ORF amplication	ATGGAGTTTACGAGTTTTCATC	TTACTCTTCCTTCAACATATG	960
	qRT-PCR	TTCTCAGCACCATTCACTCC	TCCACTTTCGACGTCACATAC	141
BcWRKY69	ORF amplication	ATGCACCATAGAGGAATTCAAG	TCATCTAGAACTATCACAGA	849
	qRT-PCR	CGGAGAGGAAGATGAATCACTG	AAGTCGCATCTCTGTCGTTG	111
BcWRKY70	ORF amplication	ATGGATATTGCTTGTAATAAC	CTAAACCGAAAACTGCTGC	852
	qRT-PCR	ATCTCCCCGTCTGCTCTAG	GACTATCGCCAGAATCCTCAAG	91
BcWRKY71	ORF amplication	ATGGCTGATCACAAGAACACTTC	TCAAGATTCGTGCTTCAAAAGCAT	1047
	qRT-PCR	TGAAAATGGTCTGCTCCGTAG	CCCACCGACTGATAATTGACC	87

BcWRKY72	ORF amplication	ATGGATCTTACAGGTATCCACG	CTAGCTTTTCTCTTCCTTGTTCACG	2529
	qRT-PCR	AGTATGCCTTCAACTTCTGGG	GCGTACAGTGGATAAAGAGGTG	147
BcWRKY74	ORF amplication	ATGGCCGTTGATCTAATGCG	TCATGCCGAAGCAAACACCA	969
	qRT-PCR	CCGTTGATCTAATGCGTTTCC	GTGTTCCATGCTCTGTAGACC	86
BcWRKY75	ORF amplication	ATGGTGAATCTAGATACGTCAC	TCATTTCCTATCCTTTTCATTG	1062
	qRT-PCR	CAGTGGGTTGTGGAGTGAAG	ATGGTTGAGGATTGGTGACG	146
BcGAPDH	qRT-PCR	CAGCGAGCAGTGAAACGGTTGA	TGACAGGAAGCCCAGCACCAA	171
