# Supplementary figures and images for: Characterization and co-expression analysis of WRKY orthologs involved in responses to multiple abiotic stresses in Pak-choi (Brassica campestris ssp. chinensis)
Source: BMC Plant Biol. 2013 Nov 25;13:188. doi: 10.1186/1471-2229-13-188 (PMC4222839; doi:10.1186/1471-2229-13-188)

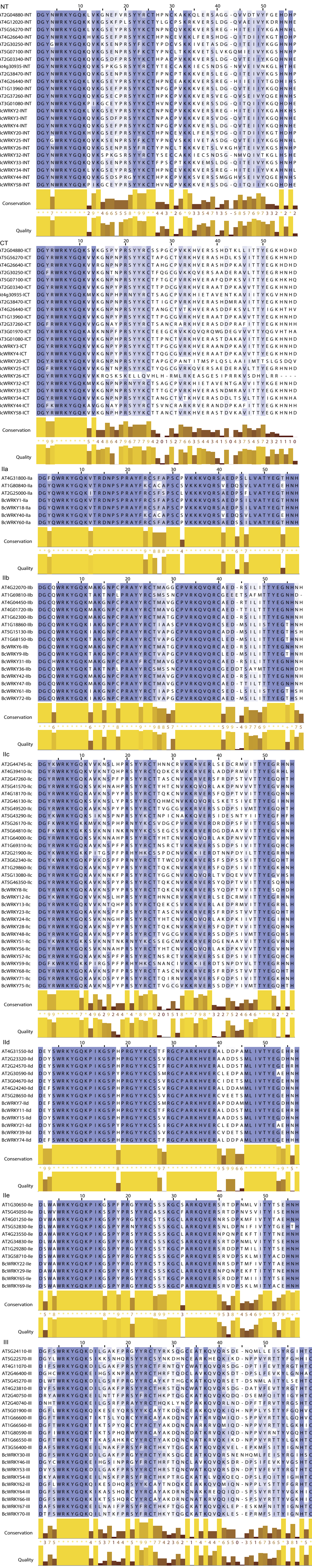

Figure S1 Alignment of WRKY domains between Pak-choi and Arabidopsis.

Supplement: Additional file 2: Figure S1 — Alignment of WRKY domains between Pak-choi and Arabidopsis. [file 1471-2229-13-188-S2.pdf]

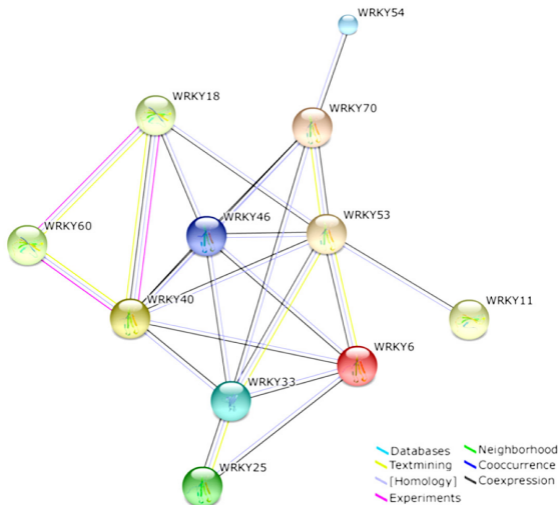

Figure S2 A confidence co-regulatory network of WRKY orthologs in Arabidopsis.

Supplement: Additional file 4: Figure S2 — A confidence co-regulatory network of WRKY orthologs in Arabidopsis. [file 1471-2229-13-188-S4.pdf]
