# Supplementary material for: Characterization and co-expression analysis of WRKY orthologs involved in responses to multiple abiotic stresses in Pak-choi (Brassica campestris ssp. chinensis)
Source: BMC Plant Biol. 2013 Nov 25;13:188. doi: 10.1186/1471-2229-13-188 (PMC4222839; doi:10.1186/1471-2229-13-188)
Supplement: Additional file 3: Table S3 — Pearson correlation coefficient of stress-induced BcWRKYs. [file 1471-2229-13-188-S3.pdf]

Supplementary Table S3 Pearson correlation coefficient of stress-induced*BcWRKY*s

Gene1	*Gene2*	*Cerrelation coefficient*
BcWRKY11	*BcWRKY13*	*1.000***
BcWRKY22	*BcWRKY26*	*0.695***
BcWRKY64	*BcWRKY75*	*0.695***
BcWRKY25	*BcWRKY33*	*0.680***
BcWRKY53	*BcWRKY54*	*0.676***
BcWRKY53	*BcWRKY70*	*0.656***
BcWRKY11	*BcWRKY64*	*0.644***
BcWRKY13	*BcWRKY64*	*0.644***
BcWRKY46	*BcWRKY40*	*0.627***
BcWRKY11	*BcWRKY75*	*0.616***
BcWRKY13	*BcWRKY75*	*0.616***
BcWRKY54	*BcWRKY75*	*0.603***
BcWRKY2	*BcWRKY54*	*0.598***
BcWRKY33	*BcWRKY46*	*0.597***
BcWRKY8	*BcWRKY75*	*0.580***
BcWRKY33	*BcWRKY75*	*0.575***
BcWRKY6	*BcWRKY40*	*-.571***
BcWRKY25	*BcWRKY40*	*0.566***
BcWRKY33	*BcWRKY53*	*0.562***
BcWRKY46	*BcWRKY70*	*0.551***
BcWRKY54	*BcWRKY64*	*0.550***
BcWRKY33	*BcWRKY70*	*0.546***
BcWRKY53	*BcWRKY75*	*0.546***
BcWRKY33	*BcWRKY40*	*0.541***
BcWRKY53	*BcWRKY64*	*0.540***
BcWRKY6	*BcWRKY11*	*0.528***
BcWRKY6	*BcWRKY13*	*0.528***
BcWRKY33	*BcWRKY54*	*0.520***
BcWRKY21	*BcWRKY39*	*0.507***
BcWRKY23	*BcWRKY33*	*0.503**
BcWRKY23	*BcWRKY46*	*0.503**
BcWRKY23	*BcWRKY34*	*0.501**
BcWRKY18	*BcWRKY26*	*0.495**
BcWRKY23	*BcWRKY70*	*0.493**
BcWRKY18	*BcWRKY70*	*0.482**
BcWRKY40	*BcWRKY70*	*0.479**
BcWRKY11	*BcWRKY54*	*0.471**
BcWRKY13	*BcWRKY54*	*0.471**
BcWRKY54	*BcWRKY70*	*0.461**
BcWRKY6	*BcWRKY25*	*-.439**
BcWRKY26	*BcWRKY40*	*0.438**
BcWRKY60	*BcWRKY70*	*0.433**
BcWRKY18	*BcWRKY33*	*0.429**
BcWRKY26	*BcWRKY60*	*0.427**
BcWRKY22	*BcWRKY60*	*0.425**
BcWRKY8	*BcWRKY11*	*0.424**
BcWRKY8	*BcWRKY13*	*0.424**
BcWRKY34	*BcWRKY46*	*0.424**
BcWRKY18	*BcWRKY40*	*0.420**
BcWRKY18	*BcWRKY23*	*0.419**
BcWRKY25	*BcWRKY70*	*0.419**
BcWRKY23	*BcWRKY40*	*0.418**
BcWRKY2	*BcWRKY39*	*-.411**
BcWRKY6	*BcWRKY70*	*-.404**
BcWRKY18	*BcWRKY25*	*0.402**
BcWRKY6	*BcWRKY33*	*-.399**

**. Correlation is significant at the 0.05 level (2-tailed).*

***. Correlation is significant at the 0.01 level (2-tailed).*
