# Supplementary material for: Characterization and co-expression analysis of WRKY orthologs involved in responses to multiple abiotic stresses in Pak-choi (Brassica campestris ssp. chinensis)
Source: BMC Plant Biol. 2013 Nov 25;13:188. doi: 10.1186/1471-2229-13-188 (PMC4222839; doi:10.1186/1471-2229-13-188)
Supplement: Additional file 5: Table S2 — Gatway cloning primers for subcelluar localization. [file 1471-2229-13-188-S5.pdf]

Supplementary Table S2 Gatway cloning primers for subcelluar localization

Gene name	Usage of primer	Sequence(5'-3')[a]
BcWRKY25	Forward primer (5'-3')	GGGGACAAGTTTGTACAAAAAAGCAGGCTTCATGGATATTGCTTGTA[illegible]
	Reverse primer (5'-3')	GGGGACCACTTTGTACAAGAAAGCTGGGTCAGATTCGTGCTTCAAAA
BcWRKY40	Forward primer (5'-3')	GGGGACAAGTTTGTACAAAAAAGCATGAACTACCCTTCAAACCCT
	Reverse primer (5'-3')	GGGGACCACTTTGTACAAGAAAGCTGGGTCTTTTTCGGTTCTGGTTCT

[a]Under line sequences repesent gatway adapter sequences

ATAAC
GCAT

'G
